# Supplementary figures and images for: Biodiversity of symbiotic microalgae associated with meiofaunal marine acoels in Southern Japan
Source: PeerJ. 2023 Oct 5;11:e16078. doi: 10.7717/peerj.16078 (PMC10560497; doi:10.7717/peerj.16078)

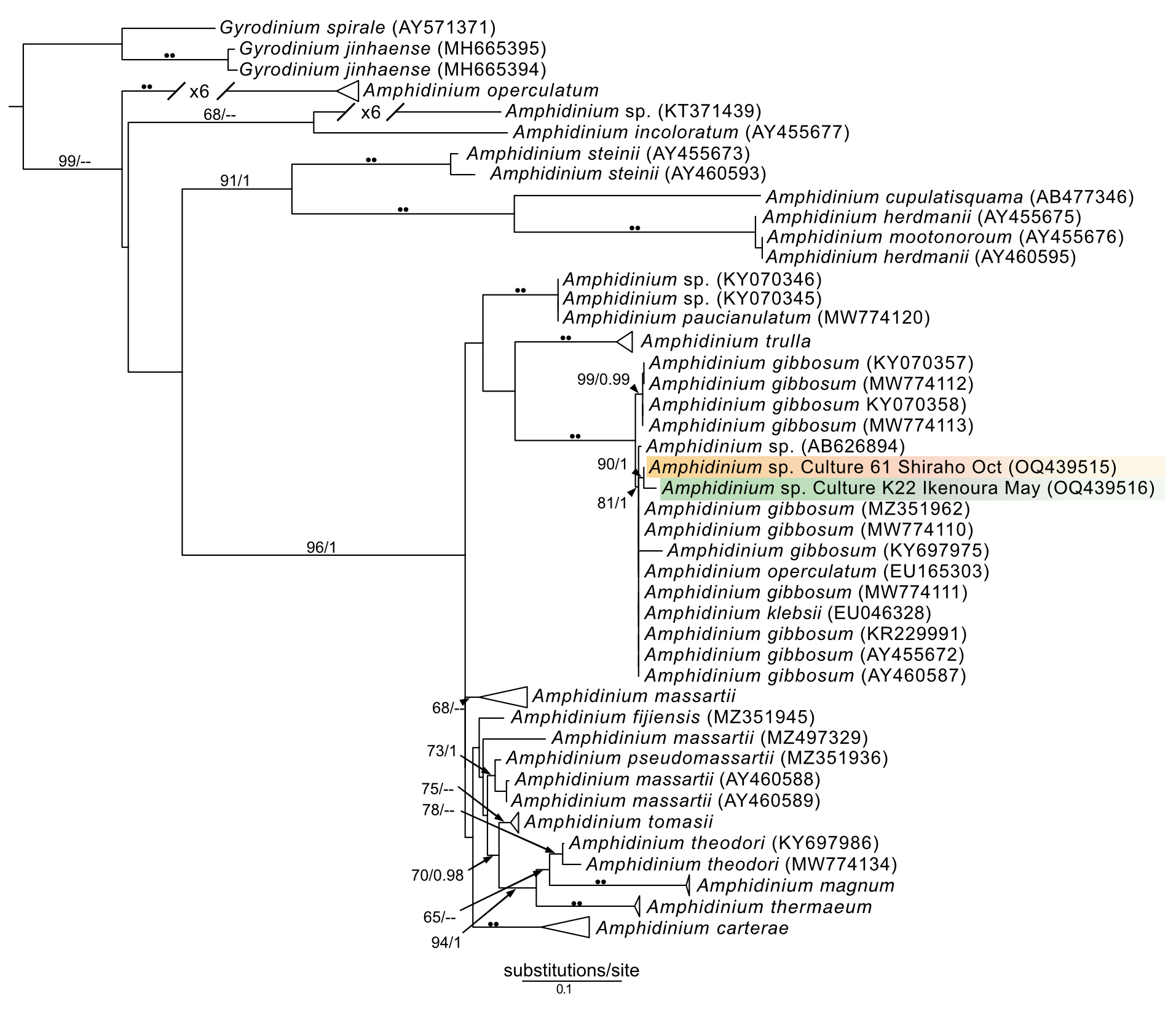

Supplement: Supplemental Information 1 — Sequences generated in this study are highlighted in colors that denote sampling times: April (blue), May (green), July (pink), and October (orange). Maximum-likelihood bootstrap values <50 and Bayesian posterior probabilities <0.95 were omitted. Black dots indicate fully supported branches (100 ML/1.00 BPP). Long branches are shortened by multiples of the substitutions/site scale bar (indicated on the branch). [file peerj-11-16078-s001.png]

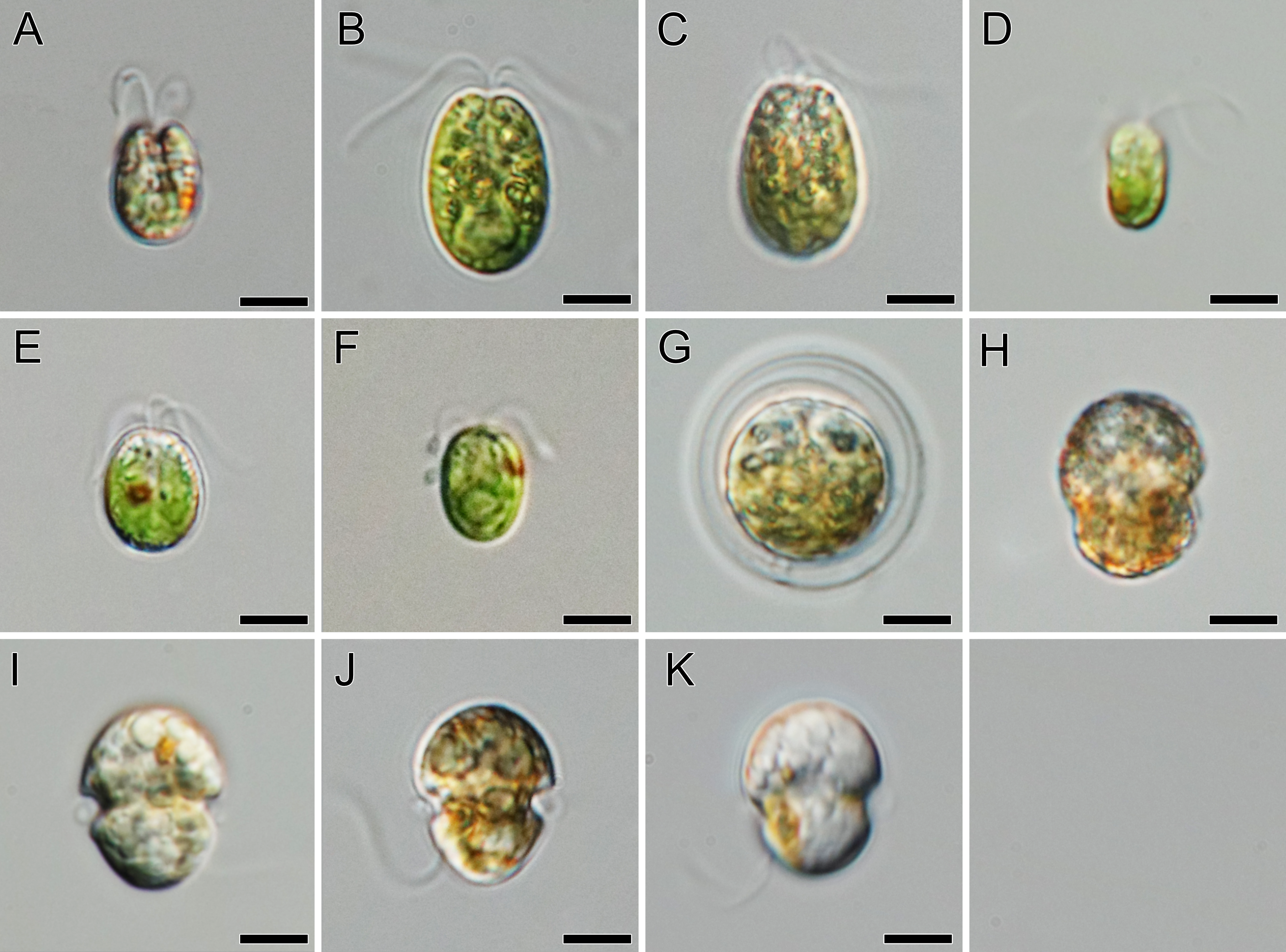

Supplement: Supplemental Information 2 — (A–G) Cultured Tetraselmis symbionts. (A) Tetraselmis established from isolate 57 collected from Kabira. (B)Tetraselmis established from isolate 35 collected from Kabira. (C) Tetraselmis established from isolate 40 collected from Kabira. (D) Tetraselmis established from isolate 31 collected from Kabira. (E) Tetraselmis established from isolate 58 collected from Shiraho. (F) Tetraselmis established from isolate K22 collected from Ikenoura. (G)Tetraselmis established from isolate 55 collected from Kabira. (H—K) Cultured Symbiodiniaceae symbionts. (H)Cladocopium established from isolate 27 collected from Kabira. (I) Symbiodinium established from isolate 66 collected from Shiraho. (J) Symbiodinium established from isolate 63 collected from Shiraho. (K) Symbiodinium established from isolate 33 collected from Kabira. Scale bars: 5 µm. [file peerj-11-16078-s002.png]

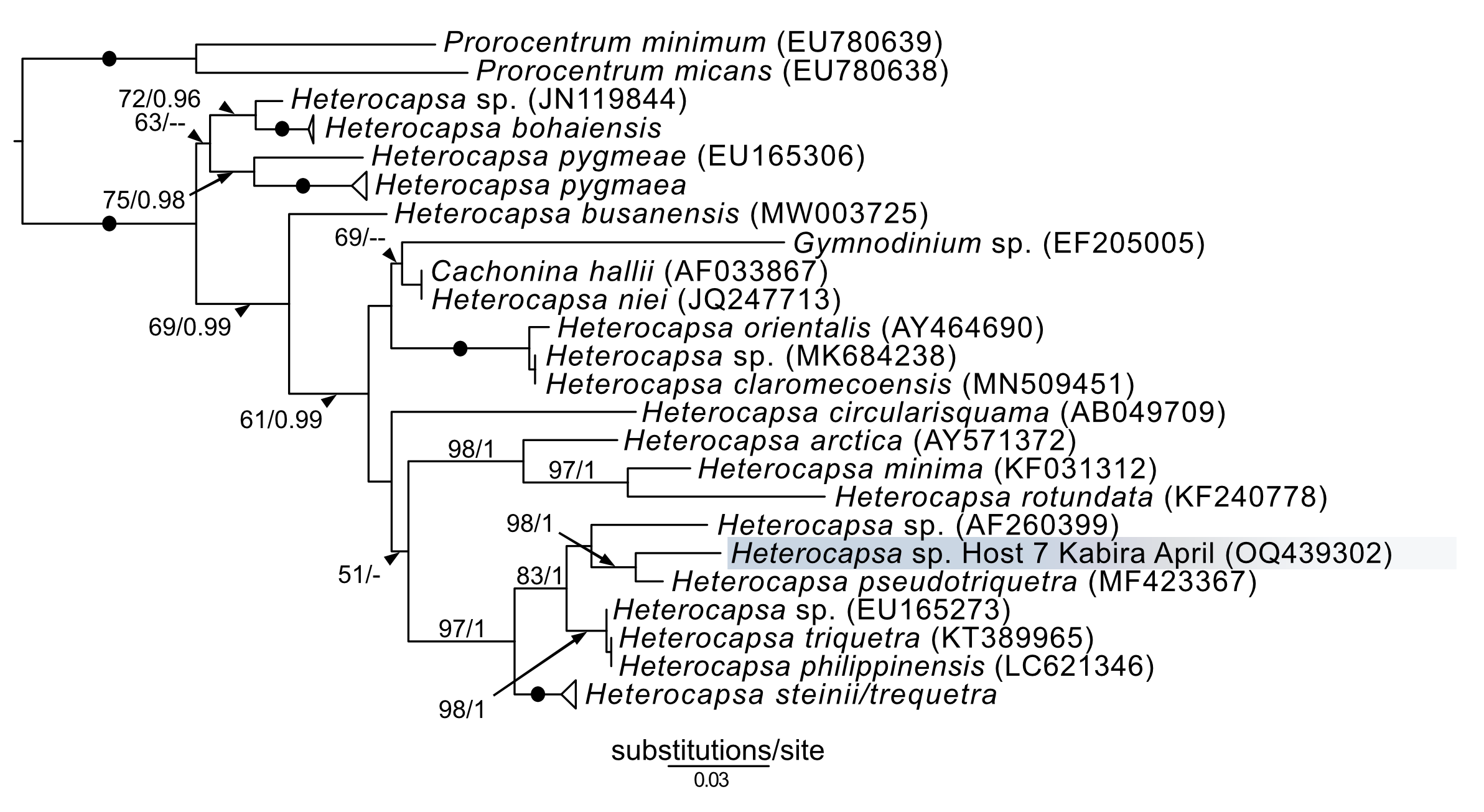

Supplement: Supplemental Information 3 — Sequences generated in this study are highlighted in blue; color is in reference to the month (April) in which the samples were collected. Maximum-likelihood bootstrap values <50 and Bayesian posterior probabilities <0.95 were omitted. Black dots indicate fully supported branches (100 ML/1.00 BPP). Long branches are shortened by multiples of the substitutions/site scale bar (indicated on the branch). [file peerj-11-16078-s003.png]
