# Supplementary material for: Biodiversity of symbiotic microalgae associated with meiofaunal marine acoels in Southern Japan
Source: PeerJ. 2023 Oct 5;11:e16078. doi: 10.7717/peerj.16078 (PMC10560497; doi:10.7717/peerj.16078)
Supplement: Supplemental Information 5 [file peerj-11-16078-s005.docx]

**Supplementary Table 2**. Primers used for PCR amplification and sequencing of 18S and 28S rRNA genes.

| Primer | Position | Sequence | Direction | Gene | Reference |
| --- | --- | --- | --- | --- | --- |
| SR1 | 1-20 | 5'-TACCTGGTTGATCCTGCCAG-3' | F | 18S | Nakayama et al., (1996) |
| SR4 | 550-568 | 5'-AGGGCAAGTCTGGTGCCAG-3' | F | 18S | Nakayama et al., (1996) |
| SR9* | 1268-1287 | 5'-AACTAAGAACGGCCATGCAC-3' | R | 18S | Nakayama et al., (1996) |
| 18SRF | 1177-1197 | 5'-CCCGTGTTGAGTCAAATTAAG-3' | R | 18S | Mo et al., (2002) |
| SR12 | 1767-1786 | 5'-CCTTCCGCAGGTTCACCTAC-3' | F | 18S | Nakayama et al., (1996) |
| TearF | 327-347 | 5'-GACATCAACCGAAGCATCAG-3' | F | 18S | This study |
| TearR | 1675-1690 | 5'-CATCCTTCGCATCGAAGAAG-3' | R | 18S | This study |
| 305F-27 | 3028-3050 | 5'-CGATAGCAAACAAGTACCATGAG-3' | F | 28S | Yamada et al. (2013) |
| D1RF1 | 2706-2725 | 5'-ACCCGCTGAATTTAAGCATA-3' | F | 28S | Takano & Horiguchi (2006) |
| 852R –70* | 3640-3658 | 5'-CGAACGATTTGCACGTCAG-3' | R | 28S | Yamada et al. (2013) |
| 28-1483R | 4203-4224 | 5'-GCTACTACCACCAAGATCTGC-3' | R | 28S | Daugbjerg et al. (2000) |

Position of the primers are based on *Toxoplasma gondii* strain RH-88 (acession number: JAAUHK010000006).

*Internal sequencing primers
